# Cutaneous Smooth Muscle Tumors: A Clinicopathological Study Focusing on the Under-Recognized Histological Features

**DOI:** 10.5146/tjpath.2019.01485

**Published:** 2020-05-15

**Authors:** Asuman Kilitci, Ömer Faruk Elmas

**Affiliations:** Department of Pathology, Ahi Evran University School of Medicine, Kırşehir, Turkey; Department of Dermatology, Ahi Evran University School of Medicine, Kırşehir, Turkey

**Keywords:** Cutaneous, Smooth muscle tumor, Piloleiomyoma, Angioleiomyoma, Leiomyosarcoma

## Abstract

***Objective:*** Cutaneous smooth muscle tumors represent a rare group of cutaneous lesions including piloleiomyoma, angioleiomyoma, genital leiomyoma, smooth muscle hamartoma, and leiomyosarcoma. In this study, we aimed to evaluate the clinical and pathological characteristics of CSMTs, focusing on the rare and unspecified histological features.

***Material and Method:*** The clinical, demographic and histological findings of the patients with CSMTs were reviewed and evaluated retrospectively. The histopathological sections were re-evaluated for all cases.

***Results:*** A total of 32 patients with CSMTs were enrolled. The majority were female (n=20). The most common tumor diagnosed was angioleiomyoma (n=19, 59.4%) followed by piloleiomyoma (n=8, 25%), smooth muscle hamartoma (n=2, 6.3%), leiomyosarcoma (n=2, 6.3%), and genital leiomyoma (n=1, 3%). Five lesions were painful and only 3 specimens were submitted with the preliminary diagnosis of a cutaneous smooth muscle tumor.

***Conclusion:*** There are very few studies investigating both clinical and histological characteristics of CSMTs in detail. Along with the classical histological clues, evaluation of the clinical findings and less-defined histological features may enhance the diagnostic accuracy. To the best of our knowledge, this study represents the first original study focusing on the clinical and pathological aspects of CSMTs in our country.

## INTRODUCTION

Cutaneous smooth muscle tumors (CSMTs) refer to a rare group of cutaneous tumors originating from piloerector smooth muscles, vascular smooth muscles or specialized soft tissues of the genital area (1,2).

Benign CSMTs mainly include piloleiomyoma (PLM), angioleiomyoma (ALM), genital leiomyoma (GLM) and smooth muscle hamartoma (SMH). PLM originates from the piloerector muscle while ALM arises from the tunica media of the vessel wall. GLM originates from the dartos muscle and nipple smooth muscle fibrils. SMH refers to the presence of congenital or acquired irregular smooth muscle bundles in the dermis (1,2). Leiomyosarcoma (LMS) describes the proliferation of smooth muscle tumors with a malignant nature (1).

There are a few original studies investigating clinicopathological features of CSMTs in the relevant literature and most of the studies covering the histopathological aspect of CSMTs are small case series or single case reports (3–10). In this study, we aimed to evaluate the clinical and pathological characteristics of benign and malignant CSMTs, focusing on the rare and unspecified histological features.

## MATERIALS and METHODS

This study was conducted at a tertiary hospital. The clinical, demographic and histological findings of the patients with CSMTs were reviewed and evaluated retrospectively. Demographic and clinical parameters such as age, site, number of lesions, preliminary diagnoses, pain status, recurrence, metastasis and sending department were obtained from the patient records. All the histological slides were re-evaluated in detail and the histomorphological findings (growth pattern, epidermal changes, presence of inflammation, degenerative changes, mitosis, necrosis, cellular atypia, presence of nerve fiber, presence of hair follicle and eccrine gland, vascular structures, lesions’ extension) were recorded. The tumor subtypes were identified, and the clinical and pathological findings were compared.

The statistical analyses conducted with the Statistical Package for Social Sciences version 21.0 software for Windows (IBM SPSS Statistics for Windows Version 21.0. Armonk, NY: IBM Corp., USA). The assumption of normality for quantitative variables was tested with the Kolmogorov-Smirnov and Shapiro-Wilk tests. The Chi-Square, Kruskal Wallis and ANOVA tests were employed for the univariate analysis of the variables in the study according to the type of the variables and the assumptions. The explanatory statistics of the variables are given as mean ± standard deviation, median, and frequencies (n). The *p* **values below 0.05 were considered to be statistically significant in all analyses.

All procedures followed the Helsinki Declaration and the study was approved by the local clinical research ethics committee (Decision no: 2019-02/26).

## RESULTS

A total of 32 patients were enrolled in the study; 20 (62.5%) of these were female and 12 were (37.5%) male. The mean ages of the female and male patients were 48.5 and 55.2 years, respectively, with no statistically significant difference. Demographic features and the mean sizes of the lesions have been summarized in Table 1.

**Table 1:** Distributions of the mean age, gender and mean tumor diameters in different subtypes of CSMTs.

**Variables**	**Angioleiomyoma**	**Piloleiomyoma**	**Smooth muscle hamartoma**	**Leiomyosarcoma**	**p**
Age (mean)	54.34±19.53	46.25±18.31	50.50±24.74	47.50±0.707	0.771
Gender (Female/Male)	13/6	4/4	2/0	0/2	0.153
Tumor diameter (mean) (cm)	1 (0.9-1.50)	1.1(0.75-1.50)	2 (1.0-3.0)	5.75 (4.5-7.0)	0.083

Pain was the only symptom reported and was present in five cases. There were no patients with a personal or familial history of cutaneous or extracutaneous leiomyomas. The most common clinical preliminary diagnosis reported was lipoma (n=4) followed by leiomyoma (n=3), sebaceous cyst (n=2), dermatofibroma (n=2), cyst (n=2), fibroma (n=1), neurofibroma (n=1), schwannoma (n=1), mycosis fungoides (n=1), morphea (n=1), bursitis (n=1), sarcoma (n=1), lymphadenopathy (n=1), hemangioma (n=1), keloidal scarring (n=1), and Bartholin gland cyst (n=1). All patients presented with solitary lesions except for a patient with multiple PLMs. In this case of multiple leiomyomas, computed tomography scans showed no evidence of concomitant visceral tumors. None of the female patients had simultaneous uterine and cutaneous leiomyomas.

The most frequent sites involved were the extremities (n=4, 71.9%) followed by the breast (n=4), back (n=2), face (n=1), vulva (n=1), and gluteal region (n=1). The most common tumor diagnosed was angioleiomyoma (n=19, 59.4%) followed by piloleiomyoma (n=8, 25%), smooth muscle hamartoma (n=2, 6.3%), leiomyosarcoma (n=2, 6.3%), and genital leiomyoma (n=1, 3%). No recurrences were determined in the follow-up visits for at least 1 year. The histological subtypes, locations and provisional diagnoses of the lesions are presented in Table 2.

**Table 2:** Clinical information of 32 patients with CSMTs.

**No**	**Histological** **Subtype**	**Location**	**Clinical information and preliminary diagnosis**
1	Angioleiomyoma	Lower extremity	Cutaneous cystic lesion
2	Angioleiomyoma	Upper extremity	Lipoma
3	Angioleiomyoma	Lower extremity	Sebaceous cyst
4	Angioleiomyoma	Upper extremity	Cutaneous mass
5	Angioleiomyoma	Upper extremity	Lipoma
6	Angioleiomyoma	Lower extremity	Palpable, mobile, cutaneous cystic mass, hard in consistency. Epidermal cyst, Dermatofibroma.
7	Angioleiomyoma	Upper extremity	Cutaneous painful mass for two years. Leiomyoma.
8	Angioleiomyoma	Upper extremity	Cutaneous painful mass for five years
9	Angioleiomyoma	Face	Cutaneous mass
10	Angioleiomyoma	Lower extremity	Prepatellar bursitis
11	Angioleiomyoma	Lower extremity	Cutaneous painful mass
12	Angioleiomyoma	Lower extremity	Lipoma
13	Angioleiomyoma	Lower extremity	Cutaneous painful nodular lesion
14	Angioleiomyoma	Lower extremity	Cutaneous mass. Lymphadenopathy, Lipoma.
15	Angioleiomyoma	Upper extremity	Cutaneous mass.
16	Angioleiomyoma	Lower extremity	Cutaneous mass.
17	Angioleiomyoma	Lower extremity	Erythematous-purple, transparent-looking nodosity. Dermatofibroma, hemangioma.
18	Angioleiomyoma	Lower extremity	Knee, cutaneous cystic mass, pain with trauma for 1.5 years
19	Angioleiomyoma	Upper extremity	Keloidal scarring.
20	Piloleiomyoma	Lower extremity	Cutaneous mass
21	Piloleiomyoma	Breast	Cutaneous mass with shrinkage of the overlying skin
22	Piloleiomyoma	Lower extremity	Cutaneous mass. Fibroma.
23	Piloleiomyoma	Lower extremity	Cutaneous mass.
24	Piloleiomyoma	Back	Neurofibroma, Leiomyoma, Schwannoma.
25	Piloleiomyoma	Breast	Cutaneous mass retracting the overlying skin
26	Piloleiomyoma	Upper extremity	Cutaneous mass
27	Piloleiomyoma	Upper extremity	Multiple, itchy, painful, erythematous cutaneous nodules for 3-4 months. Leiomyoma, Sarcoidosis.
28	Smooth muscle hamartoma	Breast	Mycosis fungoides, Morphea.
29	Smooth muscle hamartoma	Breast	Periareolar erythematous, scaly plaque 3 cm in size for three months. Bowen’s disease, squamous cell carcinoma, allergic contact dermatitis, psoriasis.
30	Leiomyosarcoma	Gluteal region	Gluteal mass
31	Leiomyosarcoma	Back	A fast-growing cutaneous solid mass on postsacral area for 6 months. Sarcoma.
32	Genital leiomyoma	Vulva	Bartholin cyst.

The mean tumor diameter was 1.6 cm, ranging from 0.75 to 7 cm. The mean diameters of the ALMs, PLMs, and SMHs showed no statistically significant difference. The largest tumor diameter was 7.0 cm in an LMS while the smallest one was 0.75 cm in a PLM. The mean sizes of each lesion subtype are shown in Table 1.

**Figure 1:**
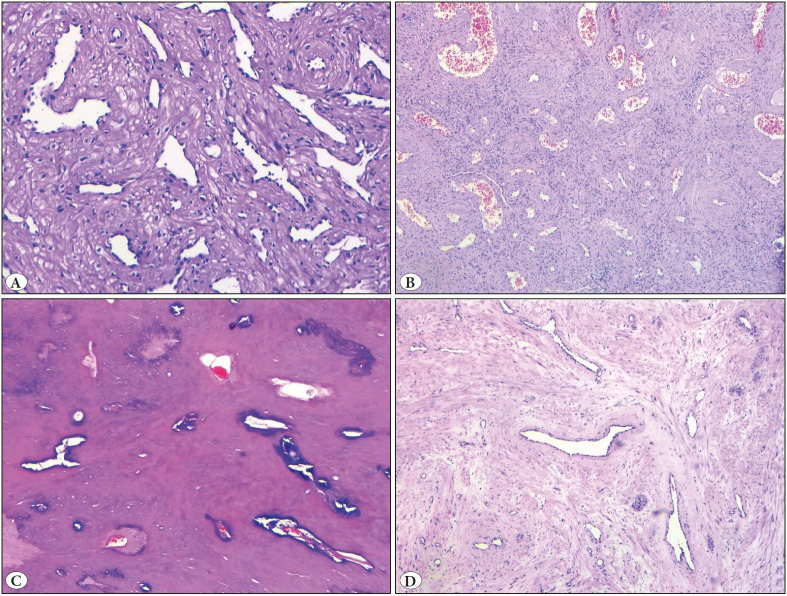
Angioleiomyoma. **A)** Thin-walled vascular channels (small veins) (H&E; x100). **B)** Thin and thick-walled vascular channels (H&E; x50). **C)** Calcification of the vascular walls and hyalinization (H&E;x50). **D)** Myxoid changes (H&E; x50).

All of the ALMs showed smooth-bordered, round and nodular growth patterns. Most of the ALMs (n=12) were limited to the lower half of the dermis while seven ALMs extended into subcutaneous fat. Four ALMs demonstrated hyalinization while two ALMs had cystic degeneration. Calcification of the vessel walls was observed in one ALM case, while another one showed myxoid degeneration. Adipocytes were also detected in an ALM. Two ALMs had mild epidermal hyperplasia while two others showed basal epidermal pigmentation. Seven ALMs had a mild chronic inflammatory cell infiltration while one showed a moderate infiltration. No hair follicles and eccrine glands involvement were observed in ALMs. The overwhelming majority of ALMs (n=15, 79%) had only thin-walled vessels while three (15.8%) ALMs had both thin- and thick-walled vessels. Only one (5.2%) ALM showed the involvement of thick-walled vessels alone (Figure 1).

**Figure 2:**
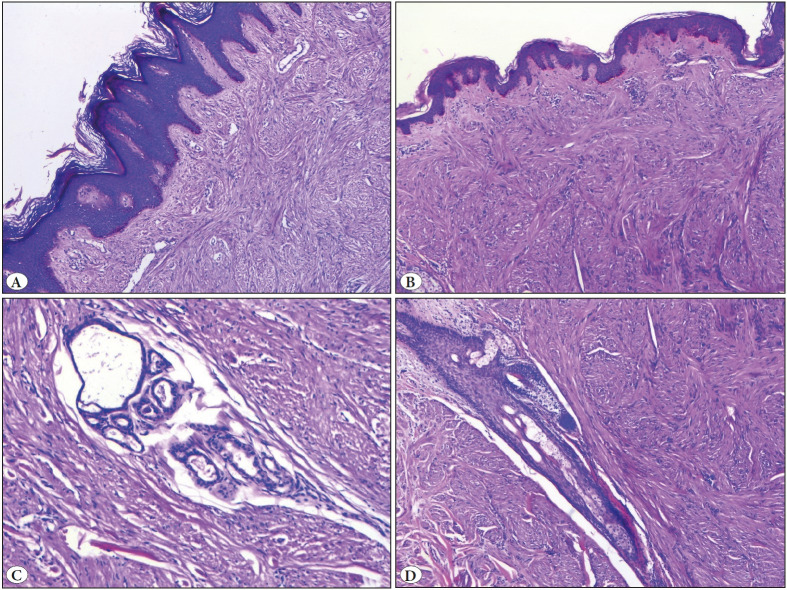
Piloleiomyoma. **A)** Epidermal hyperplasia (H&E; x50). **B)** Basal hyperpigmentation (H&E; x50).** C)** Entrapped eccrine glands (H&E; x100). **D)** Entrapped hair follicle in PLM (H&E; x50).

All PLMs demonstrated an irregular growth pattern that stretched from the upper dermis to the lower dermis. In two PLMs, muscle fibers were also observed among the rete ridges. All PLMs showed mild (n=5) or moderate (n=3) epidermal hyperplasia, while most of them (n=7) showed pigmented rete ridges. Entrapped hair follicles and eccrine glands were observed in all PLMs. Fat cells were detected in only one PLM (Figure 2). None of the ALMs or PLMs showed necrotic changes.

**Figure 3:**
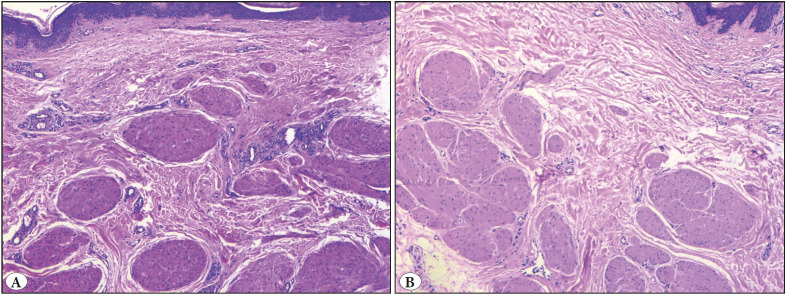
**A,B)** Smooth muscle hamartoma in two patients. Variable shape, size, and orientation of the smooth muscle bundles in the upper dermis (H&E; x50).

Both SMHs showed numerous well-defined smooth muscle bundles of varying orientation distributed throughout the dermis. While both cases had different degrees of epidermal hyperplasia, one of them also had nerve bundles (Figure 3).

**Figure 4:**
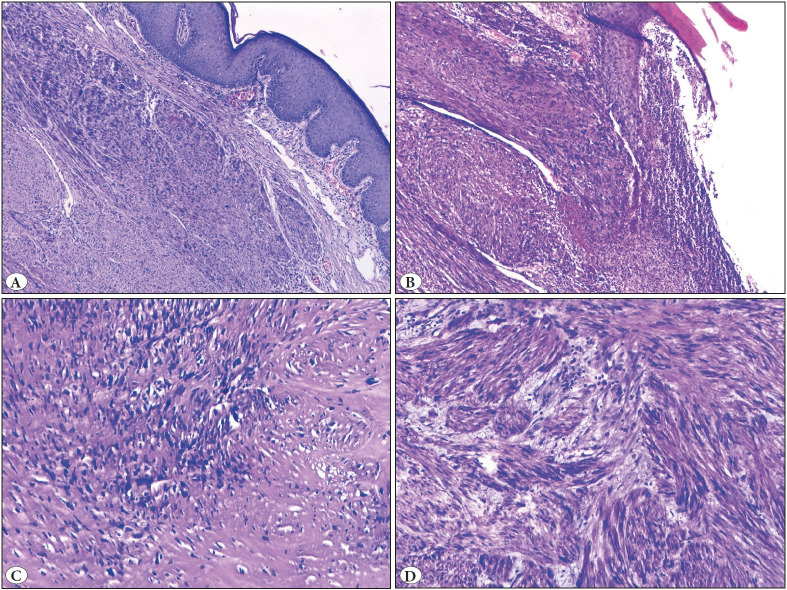
**A)** Leiomyosarcoma (H&E; x50). **B)** Ulceration (H&E; x50). **C)** Hyaline degeneration (H&E; x100). **D)** Myxoid changes (H&E; x100).

**Figure 5:**
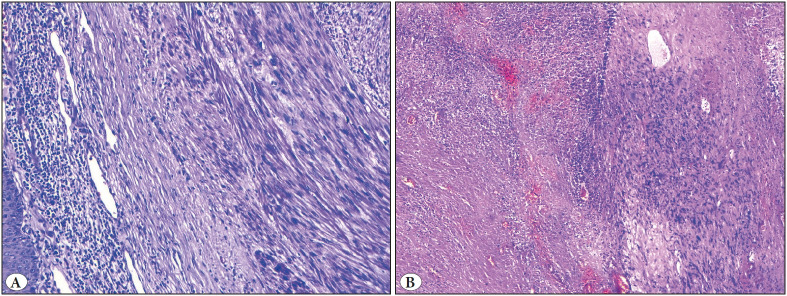
Leiomyosarcoma. **A)** Chronic inflammatory cell infiltration around the tumor (H&E; x100). **B)** Tumor necrosis (H&E; x50).

Both LMSs were characterized by a poorly circumscribed nodule comprised of a spindle cell proliferation forming rough bundles and fascicles. Necrosis was present in both LMSs while while only one of them showed atypical mitosis and ulceration (Figure 4 Figure 5). The histopathological features detected in different subtypes of CSMTs have been demonstrated in Table 3.

**Table 3:** Histological characteristics of tumor subtypes.

**Histopathological findings**	**Histopathological Diagnosis **					**p**
	**ALM (n=19)**	**PLM (n=8)**	**GLM (n=1)**	**SMH (n=2)**	**LMS (n=2)**	
**Epidermal hyperplasia**	**2**	**8**	**0**	**2**	**2**	**p=0.052**
* * Mild	2	5	0	1	1	
* * Moderate	0	3	0	1	1	
**Hyperpigmented rete ridges**	**2**	**7**	**0**	**0**	**1**	**p=0.045**
**Chronic inflammation**	**4**	**4**	**0**	**1**	**2**	**p=0.484**
* * Mild	3	4	0	0	1	
* * Moderate	1	0	0	1	1	
**Seconder changes**	**9**	**1**	**0**	**0**	**2**	**p=0.03**
* * Adipocytes	1	1	0	0	1	
* * Hyalinization	4	0	0	0	2	
* * Myxoid changes	1	0	0	0	0	
* * Calcification	1	0	0	0	0	
* * Cystic degeneration	2	0	0	0	0	
**Necrosis**	**0**	**0**	**0**	**0**	**2**	**-**
**Ulceration**	**0**	**0**	**0**	**0**	**1**	**-**
**Nerve bundles**	**8**	**2**	**0**	**1**	**1**	**p=0.0129**
* * Intratumoral	2	1	0	0	0	
* * Peritumoral	6	1	0	1	1	
**Vascular component**	**19**	**0**	**1**	**0**	**2**	**p=0.000**
* * Small arteries	1	0	0	0	0	
* * Small veins	15	0	1	0	2	
* * Small arteries + small veins	3	0	0	0	0	
**Hair follicle in lesion**	**0**	**8**	**0**	**0**	**0**	**-**
**Eccrine gland in lesion**	**0**	**8**	**0**	**0**	**0**	**-**
**Subcutaneous extension**	**7**	**4**	**0**	**0**	**1**	**p=0.105**

**ALM:** Angioleiomyoma, **PLM:** piloleiomyoma, **GLM:** Genital leiomyoma, **LMS:** Leiomyosarcoma

The immunohistochemical examination confirmed the diagnosis in all of the lesions, showing uniformly positive staining with desmin and smooth muscle actin (SMA). When comparing with desmin, SMA staining was less intense in 5 ALMs, 5 PLMs, and 2 LMSs. The remaining cases showed no remarkable differences in terms of staining patterns and intensities. In 19 cases, we used additional immunohistochemical stains (CD 31, CD34, PanCK, CD68, S100) to exclude possible differentials including fibrohistiocytic tumors, hemangiomas, and peripheral nerve sheath tumors but the results of these stains turned out to be negative, except for S100 and CD68. The last two stains showed focal-weak staining in 3 ALMs and one PLM.

A statistically significant difference was detected between histopathological findings and histopathological subtypes (p= 0.000), which has been detailed in Table 3.

## DISCUSSION

Cutaneous leiomyomas represent nearly 5 percent of all leiomyomas and are more common in adults than in children. Most of the studies have reported a male predominance (6). Unlike these studies, our study showed a slight female predominance but this difference was statistically insignificant. A higher rate of cosmetic concerns in women may explain this slight female predominance.

CSMTs may be painful possibly due to the pressure on the nerve bundles or the contraction of the muscle fibers. Pain in CSMTs has been reported as high as 54.1% of the patients in the literature (7). In our study, only 16% of the lesions were painful. Entrapped peripheral nerve sections were detected only in an ALM and two PLMs while one ALM and 6 PLMs demonstrated nerve fibers around the lesions. The paucity of the entrapped peripheral nerve fibers may explain the lower rate of pain reported in the present study. There was no statistically significant difference between pain status and tumor subtypes, gender, age, site and size of the lesions. The clinical and histopathological differential diagnoses of CSMTs should include the other painful tumors, which are dermatofibroma, eccrine spiradenoma, schwannoma, giant cell tumor of the tendon sheath, glomus tumor, hemangioma, and lipoma (11). In this study, only 3 (9.3%) patients had a preliminary clinical diagnosis of leiomyoma. The rates of an accurate preliminary diagnosis have also been reported to be low in previous studies (6,7). In this study, the most common inaccurate preliminary diagnosis was lipoma, possibly due to the subcutaneous location and consistency of the lesions. All these data may show that clinical observation has a limited contribution to the final histopathological diagnosis of CSMTs.

Extremities have been reported to be a common site for CSMTs (6,7). Our study showed a remarkably higher rate of extremity involvement (71.9%) compared to the relevant literature. The breast, back, face, vulva, and gluteal region were the other locations in this study. There was no statistically significant difference between the site of involvement and tumor subtype, gender, age, and lesions’ size.

In this study, underrecognized histological aspects of CSMTs such as epidermal features, pigmentary changes and secondary alterations have been specifically addressed. In our study, various degrees of epidermal hyperplasia were present in all PLMs while only 2 ALMs showed mild epidermal hyperplasia (p=0.052). In a study investigating the histological features of PLMs, the authors identified epidermal hyperplasia in 54.7 percent of the 53 lesions included (12). We also identified epidermal basal pigmentation in 87.5 percent of PLMs, while only two ALMs and one of the LMSs showed this finding (p=0.045). Malhotra et al. have also identified epidermal basal pigmentation in 78.4 percent of the 37 lesions included (6). Along with the previous studies, our study showed that epidermal hyperplasia and epidermal basal pigmentation may be considered as remarkable clues to PLMs.

Secondary histopathological changes such as hyalinization, myxoid changes, calcification and cystic degeneration in CSMTs have rarely been addressed in the previous reports. In the study of Ghanadan et al., hyalinization and myxoid changes were detected in 75 percent of ALMs, while none of the PLMs showed any secondary changes (7). Yokoyama et al. have identified hyalinization in 76.9 percent of 13 genital leiomyomas (13). In this study, secondary histological changes were observed in 47.3 percent of ALMs, 12.5 percent of PLMs, and both of the LMSs (p=0.003). We hypothesized that the vascular endothelial component in ALM, which is a rich source of inflammatory mediators, may cause a tendency for secondary changes (14). Duration of the lesions, traumatization, and irritation may also be associated with secondary changes.

In this study, the presence of chronic inflammatory cell infiltration was detected in 21 percent of ALMs and 50 percent of PLMs (p=0.484), suggesting the possible role of inflammation in the tumor pathogenesis. In the study of Raj et al., 86.7 percent of the cases also showed chronic inflammatory infiltration (12).

We detected entrapped hair follicles and eccrine glands in all PLMs while none of ALMs had these findings. Ghanadan et al. identified entrapped hair follicles and eccrine glands in 60 percent of 20 PLMs (7). It is clear that the presence of entrapped hair follicles and eccrine glands can be considered as a remarkable clue for PLMs.

In our study, the prominent histopathological features of LMSs were large tumor sizes, ulceration, nuclear atypia, necrosis and high count of mitosis including atypical ones. The mean mitosis count was found to be lower than 1/10 HPF for benign CSMTs. Absence of ulceration, nuclear atypia and necrosis were the other clues for a diagnosis of benign CSMT. These features were in line with the previously reported data (15). Unlike the previous studies, however, hyaline degenerative changes were observed in both LMSs.

Our study has two main limitations. The first one is the retrospective nature and the second one is the relatively small number of cases included. The fact that the patients do not apply to the clinicians because the lesion is asymptomatic or that the clinicians tend to use conservative methods instead of surgical excision may explain the small number of cases included. However, given the paucity of the studies on the subject in the relevant literature, we believe that the present study may encourage prospective studies with larger sample sizes.

In conclusion, we suggest that along with well-described histomorphological findings of CSMTs, considering the clinical features and less-defined histological characteristics may increase the diagnostic accuracy. To the best of our knowledge, this study represents the first original study focusing on the clinical and pathological aspects of CSMTs in our country.

## Conflict of Interest

The authors declare no conflict of interest.

## FUNDING

No funding to declare.
